# Pericardiectomy for Constrictive Tuberculous Pericarditis: A Systematic Review and Meta-analysis on the Etiology, Patients’ Characteristics, and the Outcomes.

**DOI:** 10.7759/cureus.18252

**Published:** 2021-09-24

**Authors:** Shikha Yadav, Suchitra Shah, Zafar Iqbal, Mohammed G Alharbi, Harjeevan S Kalra, Megha Suri, Nitin Soni, Nkiruka Okpaleke, Pousette Hamid

**Affiliations:** 1 Medicine, Kathmandu University, Kathmandu, NPL; 2 Internal Medicine, California Institute of Behavioral Neurosciences & Psychology, Fairfield, USA; 3 Emergency Medicine, California Institute of Behavioral Neurosciences & Psychology, Fairfield, USA; 4 Emergency Department, The Kidney Center, Karachi, PAK; 5 Internal Medicine, Northern Border University, Arar, SAU; 6 Internal Medicine, King Faisal Specialist Hospital and Research Centre, Riyadh, SAU; 7 Medicine-Pediatrics, California Institute of Behavioral Neurosciences & Psychology, Fairfield, USA; 8 Medicine, California Institute of Behavioral Neurosciences & Psychology, Fairfield, USA; 9 Psychiatry and Behavioral Sciences, California Institute of Behavioral Neurosciences & Psychology, Fairfield, USA; 10 Neurology, California Institute of Behavioral Neurosciences & Psychology, Fairfield, USA

**Keywords:** pericarditis, constrictive pericarditis, tuberculosis, constrictive tuberculous pericarditis, pericardiectomy

## Abstract

Tuberculosis (TB) is the most common etiology of constrictive pericarditis in the developing world. In this study, we collected currently available data to evaluate the outcomes following pericardiectomy in patients with constrictive tuberculous pericarditis. We retrieved electrical databases, including PubMed and PubMed Central, from 1985 AD and onwards. We included articles that had more than 80% TB as the etiology and articles with mixed etiologies. Pooled analysis was done in Review Manager (RevMan) version 5.2 (The Nordic Cochrane Centre, Copenhagen). and Stata Statistical Software,* *Release 16 ( StataCorp LLC, College Station, TX). We compared the mortality in patients after pericardiectomy due to TB with other etiologies. In-hospital mortality versus one-year mortality was analyzed in studies with constrictive pericarditis of mixed etiologies. We also compared pre-operative New York Heart Association (NYHA) grade to post-operative NYHA grade one year after pericardiectomy. We calculated the pooled mean of postoperative hospital stay, postoperative intensive care unit (ICU) stay, and in-hospital mortality.

A total of 12 articles and 859 patients were included in the final analysis. Pericardiectomy was performed mostly on middle-aged men with or without previous comorbidity. Total pericardiectomy was the preferred surgical procedure performed on a mean of 93% of patients. The pooled analysis shows a significant decrease in all-cause mortality in patients with TB as compared to other etiologies (pooled risk ratios (RR) 0.34 CI [0.12,1.01] I2 = 61%) and a lower but insignificant in-hospital mortality in comparison to one-year mortality in studies with mixed etiologies (RR 0.59 [0.11,3.11] I2= 61%). There was a significant improvement in the NYHA grade of the patients one year following pericardiectomy (RR 8.04, CI [5.20,12.45], I2= 0%). The mean postoperative hospital stay and the postoperative ICU stay were calculated and reported in terms of days. The mean postoperative hospital stays in studies with more than 80% of TB cases is 13.34 (10.21, 16.47) with a mean standard deviation of 4.46 (2.87, 6.05). The mean postoperative ICU stay is 1.93 (1.47, 2.39), with a mean standard deviation of 3.26 (2.51, 4.00), and the mean in-hospital mortality is 0.07 (0.02, 0.12). Similarly, the mean postoperative hospital stay in studies with mixed etiologies is 19.40 (11.93, 26.87) with a mean standard deviation of 8.26 (4.21, 12.52). The mean postoperative ICU stay is 3.52 (1.93, 5.10) with a mean standard deviation of 2.34 (1.36, 3.32). The mean in-hospital mortality is 0.06 (0.04, 0.08). There is significant heterogeneity along with a number of methodological concerns, and therefore, generalization of the data should be done with caution, and a randomized controlled trial in the future may be beneficial.

## Introduction and background

Constrictive pericarditis (CP) is a consequence of inflammation and fibrosis of the pericardium that leads to diastolic heart failure [1]. CP is a long-term complication of acute and chronic pericarditis, which can be caused by infections including tuberculosis, bacteria, and viruses, as well as malignant tumors, after cardiac surgery and radiotherapy [2]. Tuberculosis is the leading cause of constrictive pericarditis in developing countries, impacting approximately 38 - 83% of CP cases [3]. Pericardiectomy continues to be the primary treatment for patients with serious symptoms of CP. However, the perioperative mortality and lack of improvement in symptoms of heart failure are high with pericardiectomy [4,5]. Wide excision of the pericardium that extends to both the phrenic nerves and includes the diaphragmatic pericardium is defined as a complete pericardiectomy. While any pericardial excision that doesn’t meet the criteria for complete pericardiectomy is defined as an incomplete pericardiectomy [2]. There are a limited number of single-center studies on pericardiectomy for constrictive tuberculous pericarditis with no systematic review and meta-analysis to date. Here, we present a systematic review and meta-analysis on the patients’ characteristics and outcomes of patients undergoing pericardiectomy for constrictive tuberculous pericarditis and compare them with other etiologies. We also evaluated the heterogeneity and bias in the published studies.

## Review

Methods and materials

This study was conducted per the Preferred Reporting Item for Systematic Reviews and Meta-analysis (PRISMA-2020) guidelines [6]. The prospective and retrospective observational studies were initially identified from systematic reviews of the literature on the outcomes of patients who underwent pericardiectomy for constrictive pericarditis secondary to tuberculosis. We undertook updated literature searches for PubMed, and PubMed Central until June 5, 2021. Studies involving the pediatric population less than 12 years old, studies before 1985, animal studies, and studies published in languages other than English were excluded. However, those with more than 40% of TB as the etiology of constrictive pericarditis were selected. Both free-text words and MeSH subheadings were used to retrieve studies which were then screened by two independent reviewers based on their title and abstract and finally based on their full manuscript.

**Figure 1:**
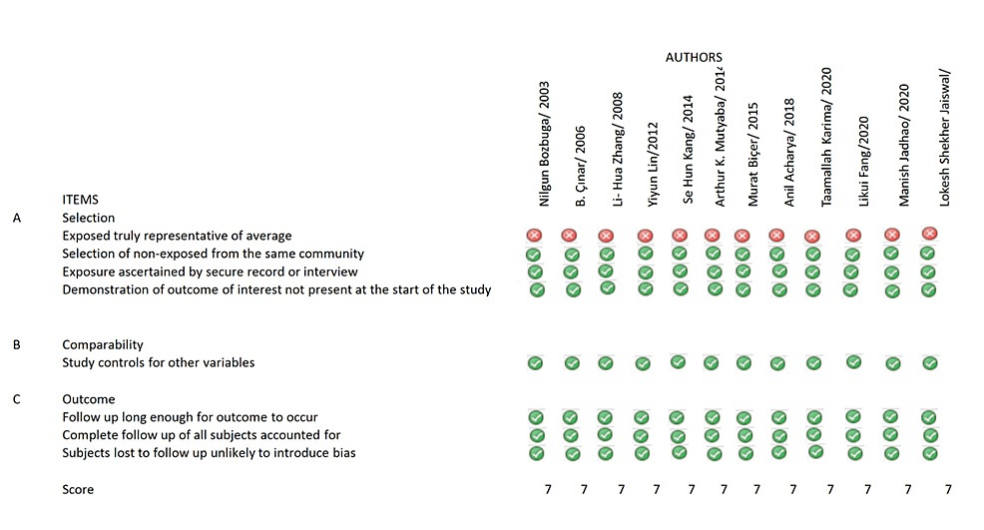
The Newcastle-Ottawa scale for evaluation of methodological quality and bias of studies.

Studies were evaluated for their methodological quality and bias with the help of the Newcastle-Ottawa scale for non-randomized studies. Two individual investigators evaluated and scored the study. Every study scored 0 or 1 for each of the items on the scale (Figure 1).

Quality Assessment of the Studies

In this study, all the data was analyzed using Review Manager (RevMan) version 5.2 (The Nordic Cochrane Centre, Copenhagen) and Stata Statistical Software, Release 16 (StataCorp LLC, College Station, TX). The reporting of continuous variables is done as mean and standard deviation (SD) or 95% confidence interval (CI), and categorical variables are reported as frequency and proportions (%). Pooled risk ratios (RRs) and corresponding confidence intervals were calculated using a random-effects model as per DerSimonian-Laird. The Cochrane’s Q test and the I^2^ test were used to assess the heterogeneity between the studies. I^2^ greater than 50% and p-value < 0.10 for the Chi-square test indicate significant heterogeneity. A statistical test for publication bias was done using Beggs regression asymmetry test, and a funnel plot was used to visualize publication bias amongst the studies used for meta-analysis. 

The outcomes were presented as pooled mean prevalence and 95% CI. We also calculated the pooled mean postoperative ICU stay and hospital stay in patients with TB and mixed causes of pericarditis. The mean postoperative hospital stay and the postoperative ICU stay are calculated and reported in terms of days. We compared the all-cause mortality in patients with constrictive tuberculous pericarditis to other etiologies such as idiopathic and post-cardiac surgery. We also compared in-hospital mortality with one-year mortality in patients with constrictive pericarditis.

Results

Search Results

The PubMed search showed 17,260 articles, and PubMed Central showed 6,144 articles with the help of the above-mentioned keywords (June 5, 2021). We identified 1200 articles after implementing the inclusion/exclusion criteria. The articles were then screened by two investigators based on the title. Furthermore, one investigator screened the articles based on the full texts. PubMed search results, with regular keywords, are demonstrated in Table 1.

**Table 1:** Search results on PubMed with regular keywords.

Total studies	17, 260
Studies after 1985 AD and onwards	10,339
Studies in the English Language	2,745
Journal Articles	2,186
Meta-Analysis	11
Observational Study	19
Randomized Controlled Trial	23
Systematic Review	25

**Figure 2:**
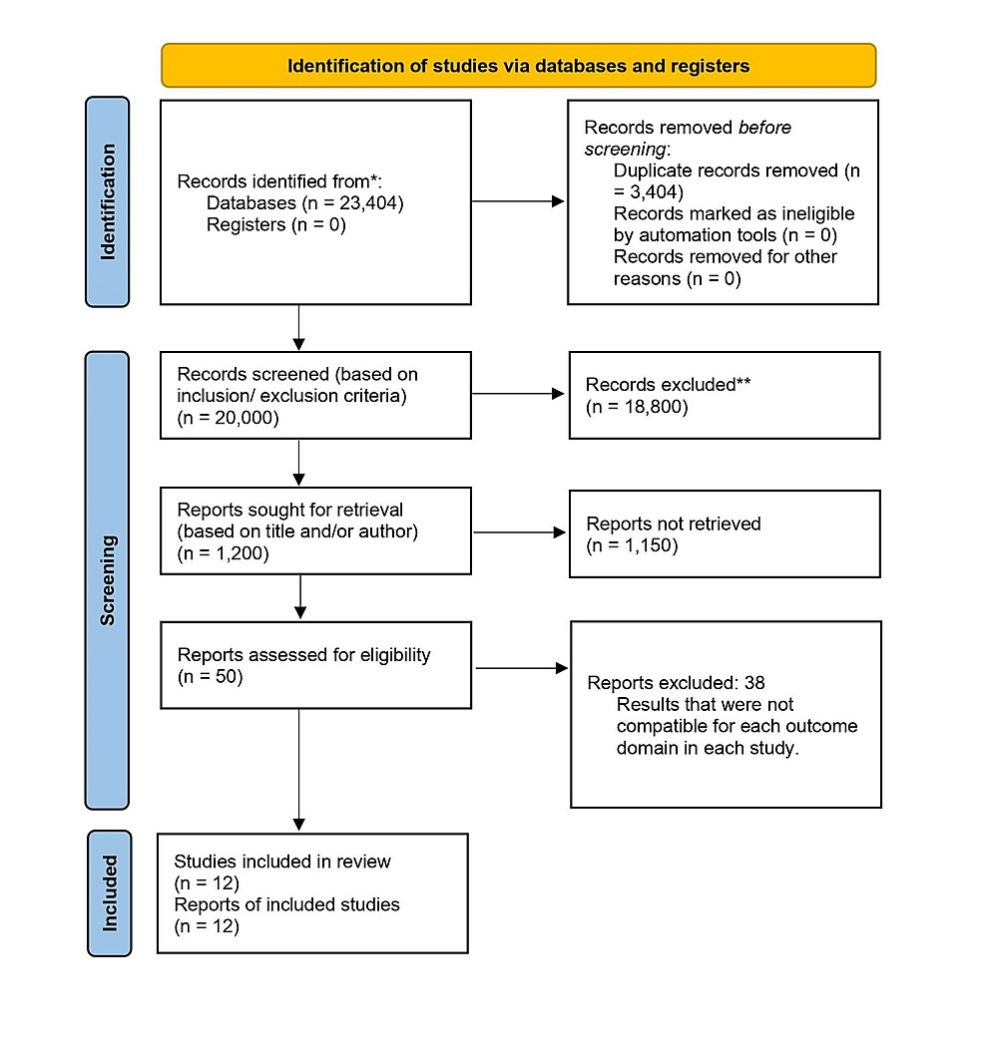
PRISMA flow diagram-2020 for systematic reviews which include searches of databases and registers only. PRISMA - Preferred Reporting Item for Systematic Reviews and Meta-analysis; n - number of articles

PRISMA flow diagram for the results obtained is shown in Figure 2. Six recent retrospective observational studies were identified with more than 80% of tuberculosis patients and the other six studies had mixed etiologies. A total of 859 patients were analyzed for pre-operative patients’ characteristics and the outcomes of patients following pericardiectomy. All the studies are retrospective cohort studies and were published from 2000 to 2021.

Patients Characteristics 

Table 2 displays the patients’ characteristics of constrictive tuberculous pericarditis. The studies overall described 859 patients, of whom 471 patients had only tuberculosis as the etiology of constrictive pericarditis, and 388 patients had mixed etiologies. The studies are mostly from the developing parts of the world. The majority of the patients in the study were males within the age group of 17 to 79 years.

**Table 2:** Patients’ characteristics of the studies No. of Pts.: Number of patients in the study, Male %: Percentage of males in the study, % of Tuberculosis: Percentage of tuberculosis cases within the study, % of idiopathic: Percentage of idiopathic cases within the study, % of other causes: Percentage of other causes of constrictive pericarditis, N/A: Not available.

Author/Year of Publication	Study origin	Type of study	Duration of the study	No. of Pts.	Gender (Male%)	Mean age (years)	% of Tuberculosis	% of Idiopathic	% of other causes
Bozbuga et al./ 2003 [7]	Turkey	Retrospective observational	1985 - 2002	36	28	32.2+/- 16.3	100	-	-
Çınar et al./ 2006 [3]	Turkey	Retrospective observational	1990 -2005	82	70	17 - 79	88	-	12
Zhang et al./ 2008 [8]	China	Retrospective observational	2000- 2007	150	-	-	78.7	N/A	21.3
Lin et al./2012 [9]	China	Retrospective observational	2005- 2011	51	75	40.1 +/- 15.5	65	25	10
Kang et al./ 2014 [2]	Korea	Retrospective observational	1996-2010	85	61.2	51.8 +/- 13.7	42.4	57.6	-
Mutyaba et al./ 2014 [10]	Cape Town	Retrospective observational	1990- 2012	121	65.3	41.3 +/- 16.1	91	4.9	3.1
Biçer et al./ 2015 [11]	Turkey	Retrospective observational	1992 - 2014	47	64	45.8 +/-16.7	46.8	31.9	21.3
Acharya et al./ 2018 [12]	Nepal	Retrospective observational	2003 - 2013	130	71	22.95 +/-12.55	100	-	-
Karima et al./ 2020 [13]	Tunisia	Retrospective observational	1994- 2017	25	80	40.46+/-16.74	44	N/A	56
Fang et al./ 2020 [14]	China	Retrospective observational	2012-2019	88	-	54.6 +/- 16.8	100	-	-
Jadhao et al./ 2020 [15]	India	Retrospective observational	2013- 2018	30	60	32 +/-15.5	50	37	13
Jaiswal et al./ 2021 [16]	Nepal	Retrospective observational	2015 - 2018	14	72	38 +/- 13.3	100	-	-

*Graphical Representation of the Results* 

**Figure 3:**
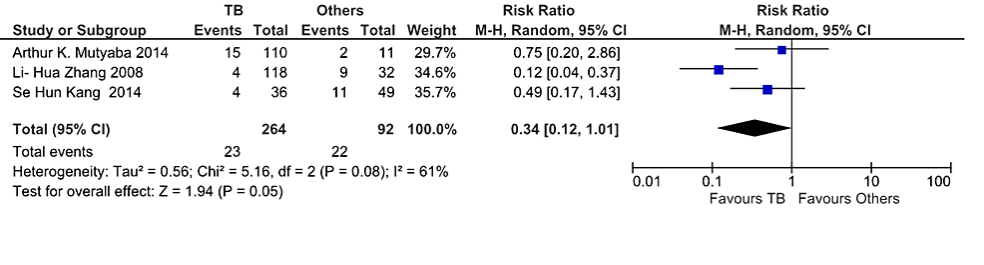
All-cause mortality after pericardiectomy in patients with constrictive tuberculous pericarditis and other etiologies. TB Events: Mortality after pericardiectomy in patients with constrictive pericarditis with tuberculosis as etiology. Other Events: Mortality after pericardiectomy in patients with constrictive pericarditis with etiologies other than tuberculosis. The Cochran-Mantel-Haenszel method and the random-effects model were used to calculate the pooled risk ratio. M-H - Mantel-Haenszel Test; Tau^2^ - Tau-squared test for random effects model; Chi^2^ - Chi-squared test; df - degree of freedom; I^2^ - I^2^ test for heterogeneity; Z - Standard score References [2,8,10]

Forest plot to compare TB with other etiologies: Pooled data from the three studies (Figure 3) show all-cause mortality in 23 out of 264 patients with TB as etiology and 22 out of 92 patients with etiologies other than TB who underwent pericardiectomy for constrictive pericarditis. The pooled result shows a significant decrease in mortality in patients with TB as compared to other etiologies (RR 0.34, CI [0.12, 1.01], I2 = 61%). Mutyaba et al. [10], Zhang et al. [8], and Kang et al. [2] are the studies evaluated in the forest plot.

**Figure 4:**
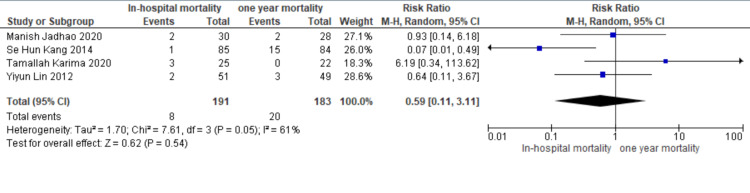
In-hospital mortality versus one-year mortality post-pericardiectomy in patients with constrictive pericarditis. In-hospital mortality events: Mortality in patients within 30 days of pericardiectomy for constrictive pericarditis. One-year mortality Events: Mortality in patients within one year of pericardiectomy for constrictive pericarditis. The Cochran-Mantel-Haenszel method and the random-effects model were used to calculate the pooled risk ratio. M-H - Mantel-Haenszel Test; Tau^2^ - Tau-squared test for random effects model; Chi^2^ - Chi-squared test; df - degree of freedom; I^2^ - I^2^ test for heterogeneity; Z - Standard score References [2,9,13,15]

Forest plot to compare in-hospital mortality with one-year mortality: Pooled data from four studies (Figure 4) show in-hospital mortality in eight out of 191 patients and one-year mortality in 20 out of 183 patients. The pooled result shows lower but insignificant in-hospital mortality in comparison to one-year mortality in patients undergoing pericardiectomy for constrictive pericarditis (RR 0.59, CI [0.11,3.11], I2= 61%). Jadhao et al. [15], Kang et al. [2], Karima et al. [13], Lin et al. [9] are the studies evaluated in the forest plot.

**Figure 5:**
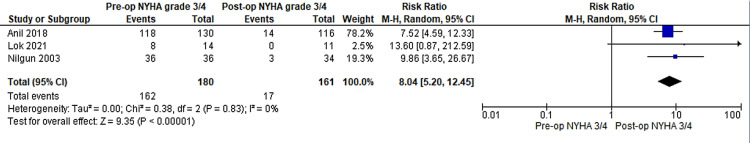
Pre-operative NYHA grade III and IV versus post-operative NYHA grade III and IV after one year in patients with constrictive tuberculous pericarditis. Pre-op NYHA grade 3/4: Pre-operative New York Heart Association (NYHA) grades III and IV in patients with constrictive tuberculous pericarditis. Post-op NYHA grade 3/4: Post-operative NYHA grades III and IV after one year in patients with constrictive tuberculous pericarditis. The Cochran- Mantel- Haenszel method and the random-effects model were used to calculate the risk ratio. M-H - Mantel-Haenszel Test; Tau^2^ - Tau-squared test for random effects model; Chi^2^ - Chi-squared test; df - degree of freedom; I^2^ - I^2^ test for heterogeneity; Z - Standard score References [7,12,16]

Forest plot to compare the symptomatic outcome: Pooled data from the three studies (Figure 5) shows 162 out of 180 patients had a pre-operative New York Heart Association (NYHA) grade of III and IV, which significantly improved to 17 out of 161 patients with an NYHA grade of III and IV (RR 8.04, CI [5.20, 12.45], I2=0%) one year after pericardiectomy in patients with constrictive tuberculous pericarditis. Acharya et al. [12], Jaiswal et al. [16], Bozbuga et al. [7] are the studies used to evaluate the symptomatic outcome in terms of NYHA grade postoperatively in patients with constrictive tuberculous pericarditis.

Postoperative Hospital Stay

**Figure 6:**
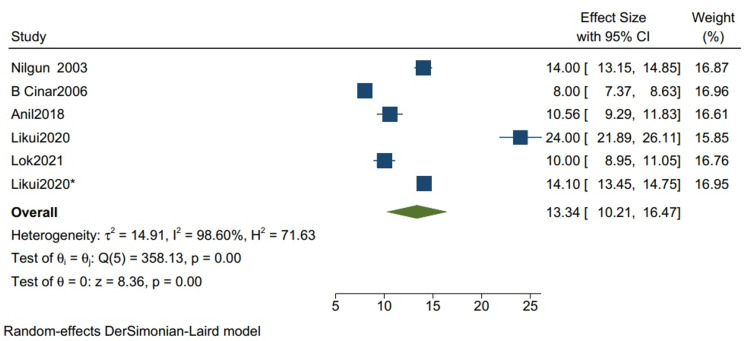
Mean of postoperative hospital stay for studies with more than 80% of TB cases. Random-effects DerSimonian-Laird model was used to calculate the pooled mean. τ^2 ^- Hotelling's t-squared statistic; H^2 ^- H^2^ test; θ - Vector of parameters of a probability distribution; τ: Between study variance References [3,7,12,14,16]

**Figure 7:**
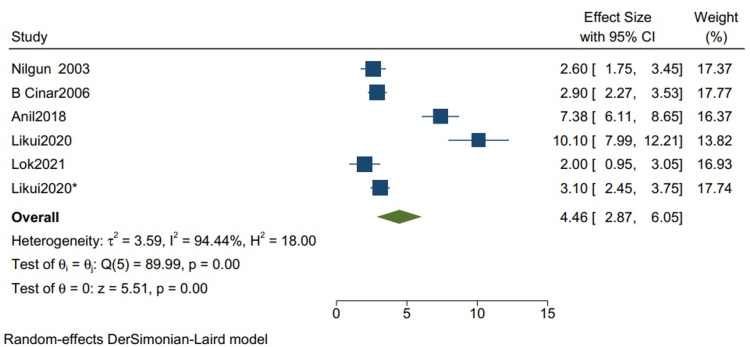
Mean of the standard deviation for the postoperative hospital stay for studies with more than 80% of TB cases. Random-effects DerSimonian-Laird model was used to calculate the pooled mean standard deviation. τ^2^ - Hotelling's t-squared statistic; H^2^ - H^2^ test; θ - Vector of parameters of a probability distribution; τ: Between study variance; I^2^ - I^2^ test for heterogeneity References [3,7,12,14,16]

The outcome in studies with more than 80% TB cases: The mean postoperative hospital stays in studies with more than 80% TB cases is 13.34 (10.21, 16.47) (Figure 6) with a mean standard deviation of 4.46 (2.87, 6.05) (Figure 7). Nilgun 2003: Bozbuga et al. [7], B Cinar2006: Cinar et al. [3], Anil2018: Acharya et al. [12], Likui2020: Fang et al. [14], Lok2021: Jaiswal et al. [16] are the studies used to evaluate the outcome in studies with more than 80% TB cases.

Postoperative ICU Stay

**Figure 8:**
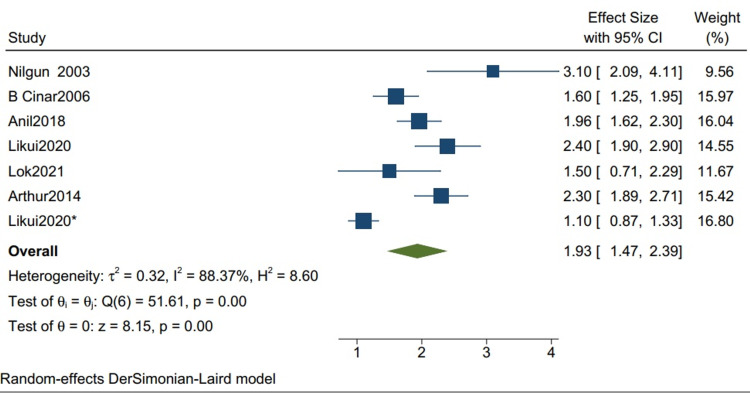
Mean of postoperative ICU stay in studies with more than 80% of TB cases. Random-effects DerSimonian-Laird model was used to calculate the pooled mean. τ^2^ - Hotelling's t-squared statistic; H^2^ - H^2^ test; θ - Vector of parameters of a probability distribution; τ: Between study variance; I^2^ - I^2^ test for heterogeneity References [3,7,10,12,14,16]

**Figure 9:**
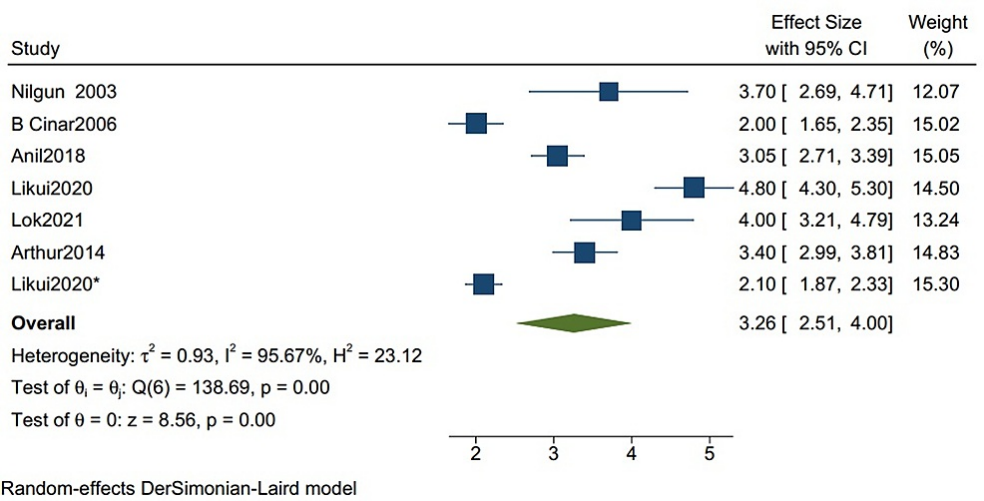
The mean standard deviation for postoperative ICU stay for studies with more than 80% of TB cases. Random-effects DerSimonian-Laird model was used to calculate the pooled mean. τ^2^ - Hotelling's t-squared statistic; H^2^ - H^2^ test; θ - Vector of parameters of a probability distribution; τ: Between study variance; I^2^ - I^2^ test for heterogeneity References [3,7,10,12,14,16]

The mean postoperative ICU stay in studies with more than 80% TB cases is 1.93 (1.47, 2.39) (Figure 8), with a mean standard deviation of 3.26 (2.51, 4.00) (Figure 9).

In-hospital Mortality 

**Figure 10:**
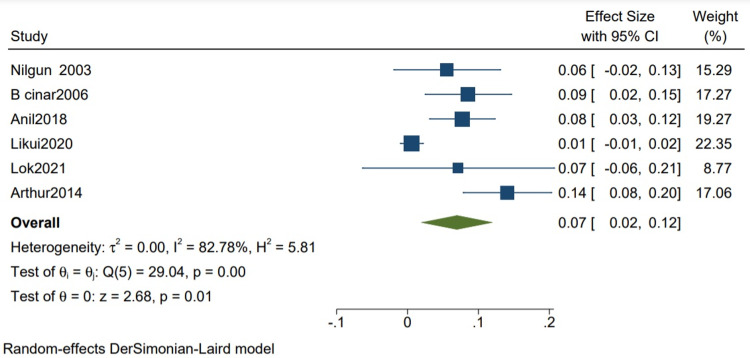
Mean in-hospital mortality in studies with more than 80% TB cases. Random-effects DerSimonian-Laird model was used to calculate the pooled mean. τ^2^ - Hotelling's t-squared statistic; H^2^ - H^2^ test; θ - Vector of parameters of a probability distribution; τ: Between study variance; I^2^ - I^2^ test for heterogeneity References [3,7,10,12,14,16]

The mean in-hospital mortality is 0.07 (0.02, 0.12) (Figure 10).

Funnel plot to access publication bias 

**Figure 11:**
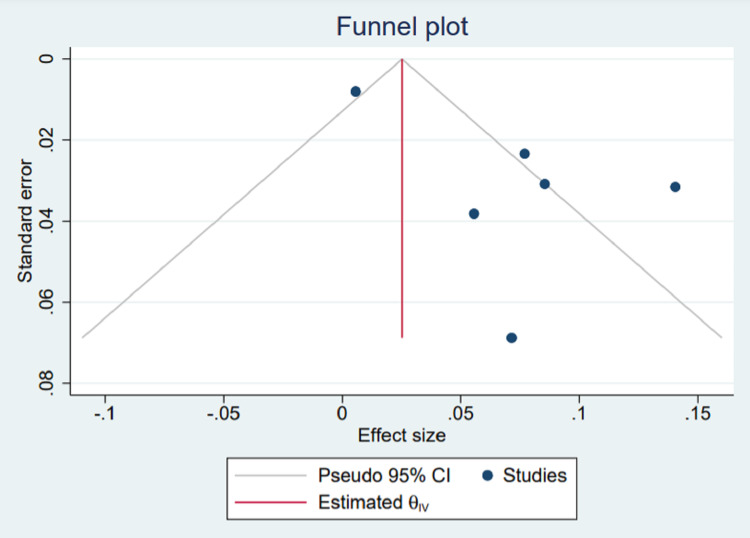
Funnel plot for studies with more than 80% of TB cases. References [3,7,10,12,14,16]

Visualization of publication bias amongst the studies with more than 80% TB was done using a funnel plot (Figure 11). The plot showed asymmetry, which is interpreted as having some kind of reporting bias or publication bias. We further assessed the asymmetry using the Beggs regression asymmetry test, and it was statistically insignificant (p = 1.29), which shows no publication bias.

The Outcome in Studies With Mixed Etiologies

**Figure 12:**
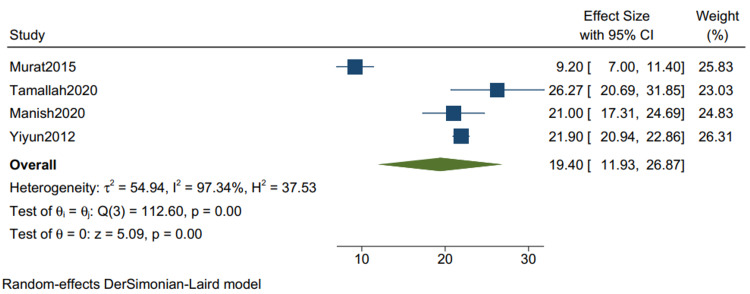
Mean of postoperative hospital stay in studies with mixed etiologies. Random-effects DerSimonian-Laird model was used to calculate the pooled mean. τ^2^ - Hotelling's t-squared statistic; H^2^ - H^2^ test; θ - Vector of parameters of a probability distribution; τ: Between study variance; I^2^ - I^2^ test for heterogeneity References [9,11,13,15]

**Figure 13:**
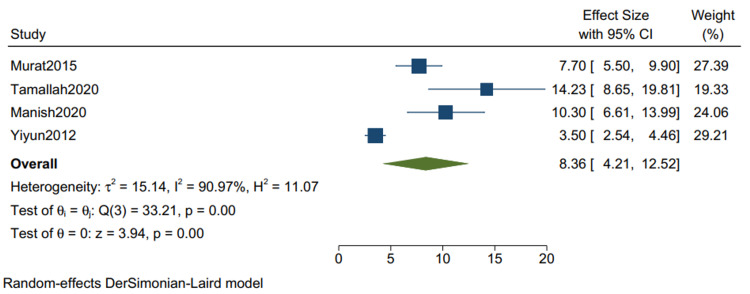
Mean of the standard deviation of postoperative hospital stay in studies with mixed etiologies. Random-effects DerSimonian-Laird model was used to calculate the pooled mean. τ^2^ - Hotelling's t-squared statistic; H^2 ^- H^2^ test; θ - Vector of parameters of a probability distribution; τ: Between study variance; I^2^ - I^2^ test for heterogeneity References [9,11,13,15]

Postoperative hospital stays: The mean postoperative hospital stays in studies with mixed etiologies is 19.40 (11.93, 26.87) (Figure 12), with a mean standard deviation of 8.26 (4.21, 12.52) (Figure 13). Murat2015: Bicer et al. [11], Tamallah2020: Karima et al. [13], Manish2020: Jadhao et al. [15], Yiyun2012: Lin et al. [9], Se Hung Kang2014: Kang et al. [2], Li: Zhang et al. [8] are the studies used to evaluate the outcomes in studies with mixed etiologies.

**Figure 14:**
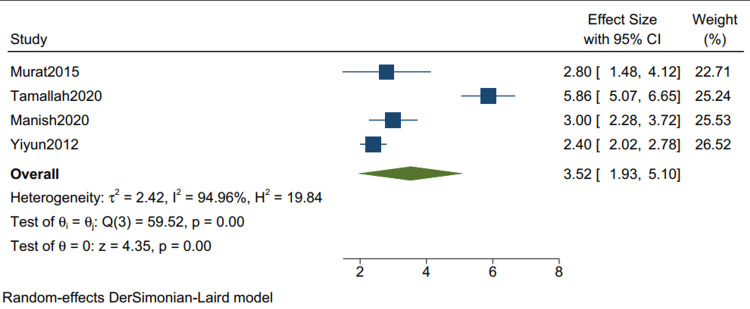
Mean of postoperative ICU stay in studies with mixed etiologies. Random-effects DerSimonian-Laird model was used to calculate the pooled mean. τ^2^ - Hotelling's t-squared statistic; H^2^ - H^2^ test; θ - Vector of parameters of a probability distribution; τ: Between study variance; I^2^ - I^2 ^test for heterogeneity References [9,11,13,15]

**Figure 15:**
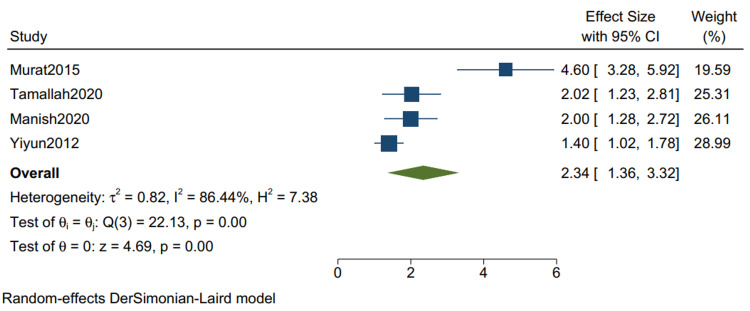
Mean of the standard deviation of postoperative ICU stay in studies with mixed etiologies. Random-effects DerSimonian-Laird model was used to calculate the pooled mean. τ^2^ - Hotelling's t-squared statistic; H^2^ - H^2^ test; θ - Vector of parameters of a probability distribution; τ: Between study variance; I^2^ - I^2^ test for heterogeneity References [9,11,13,15]

Postoperative ICU Stay*: *The mean postoperative ICU stay in studies with mixed etiologies is 3.52 (1.93, 5.10) (Figure 14), with a mean standard deviation of 2.34 (1.36, 3.32) (Figure 15).

**Figure 16:**
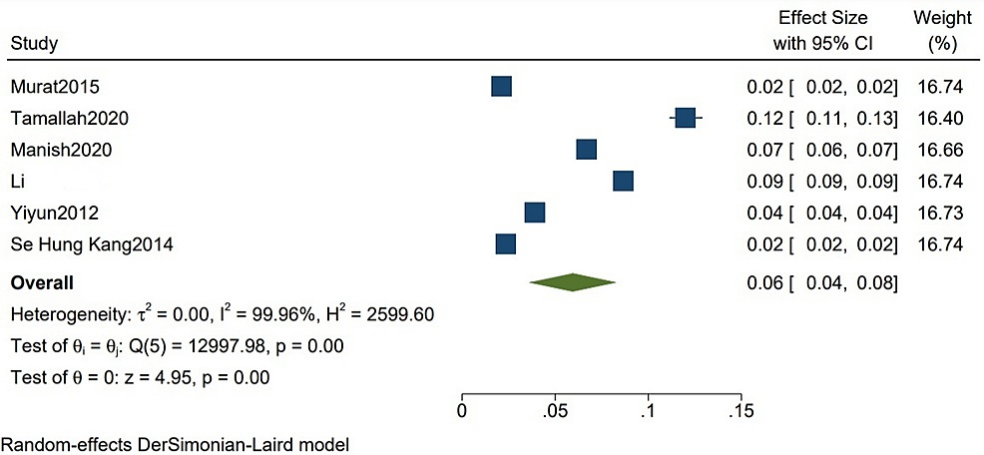
Mean in-hospital mortality in studies with mixed etiologies Random-effects DerSimonian-Laird model was used to calculate the pooled mean. τ^2^ - Hotelling's t-squared statistic; H^2^ - H^2^ test; θ - Vector of parameters of a probability distribution; τ: Between study variance; I^2^ - I^2^ test for heterogeneity References [2,8,9,11,13,15]

In-hospital Mortality: The mean in-hospital mortality is 0.06 (0.04, 0.08) (Figure 16).

**Figure 17:**
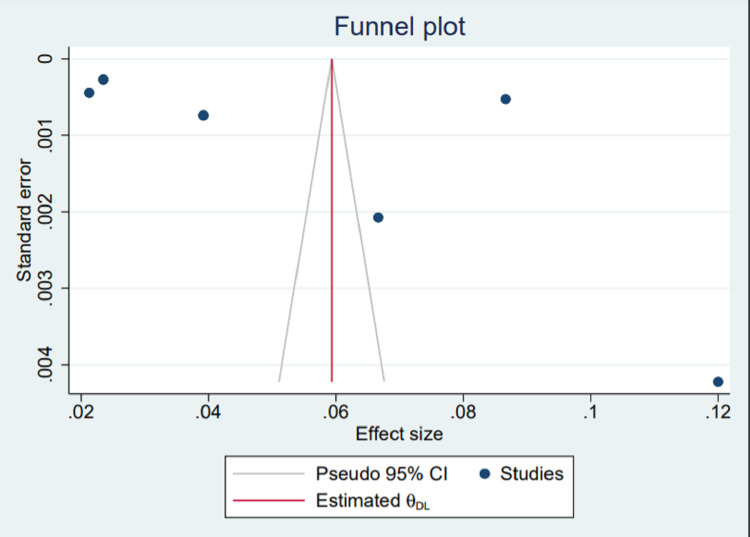
Funnel plot for studies with mixed etiologies. References [8,9,11,13,15]

Funnel plot to access publication bias: Visualization of publication bias amongst the studies with mixed etiologies was done using a funnel plot (Figure 17). The plot showed asymmetry, which is interpreted as having some kind of reporting bias or publication bias. We further assessed the asymmetry using the Beggs regression asymmetry test, and it was statistically insignificant (p = 0.1329), which shows no publication bias.

Types of Pericardiectomy

**Figure 18:**
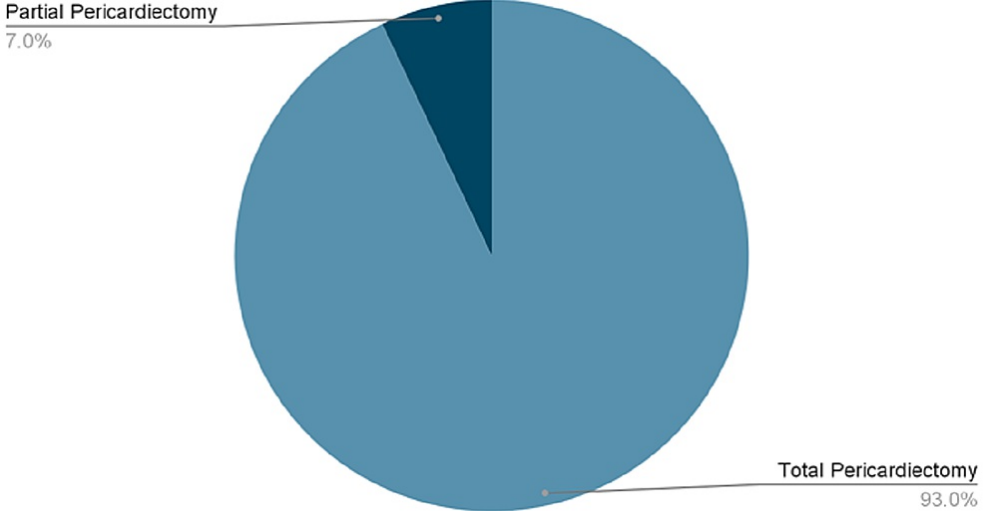
Types of pericardiectomy performed on the overall patients included in our study.

Figure 18 is a graphical representation of types of pericardiectomy performed in patients with constrictive pericarditis under our study. We observed that 93% of patients had total pericardiectomy while 7% of patients had partial pericardiectomy performed for constrictive pericarditis.

Discussion

Tuberculosis is the most common cause of constrictive pericarditis in low and middle-income areas [10,17]. In nearly 50% of the patients, there is a resolution of tuberculous pericardial effusion without any development of constriction, while the rest of the patients develop chronic constrictive pericarditis regardless of similar and adequate anti-tuberculosis treatment [18].

Patients with tuberculosis as the etiology for constrictive pericarditis received anti-tubercular therapy either before the surgery or after the surgery. In the study conducted by Acharya et al., all patients received anti-tubercular therapy (ATT) for six months (two months of ethambutol, rifampicin, isoniazid, and pyrazinamide, and four months of rifampicin and isoniazid). A new course was not initiated if the patient had previously completed the course of ATT. If ATT was not taken by a patient, the regimen was initiated only if there was strong clinical evidence of active tuberculosis or when the pericardial biopsy was positive [12].

All patients in the study underwent pericardiectomy for constrictive pericarditis. A mean of 93% of patients from all the enlisted studies underwent total pericardiectomy (Figure 18). Preoperatively, the patients presented with NYHA grade III and grade IV heart failure, ascites, peripheral edema, pleural effusion, hepatomegaly, jugular venous pressure elevation, and so on. The outcome of the patients was assessed majorly with the help of length of ICU stay, length of hospital stay, in-hospital mortality, improvement in the grade of NYHA heart failure, and long-term mortality [2,3,7,8-16]. 

Cinar et. al. in his study reported that the primary clinical presentation of patients with constrictive tuberculous pericarditis was congestive heart failure, which was seen in 90% of the patients. The symptoms were present for a median of 24 months before surgery. Out of the total, 77% of patients were in NYHA classes II and III and the remainder were in class IV. After the surgery, the mean ICU stay and hospital stays were 2+/-1.6 and 8+/-2.9 days, respectively, with perioperative mortality being 8.6% [3].

A recent meta-analysis on population characteristics and outcomes of patients undergoing pericardiectomy for constrictive pericarditis conducted by Tzani et. al. excluded studies with more than 10% TB cases. The most common etiology in their study was idiopathic. They concluded that the mortality was higher in radiation and after-cardiac surgery patients, as compared to idiopathic [2].

Our study reports a significant decrease in all-cause mortality in patients with TB as compared to other etiologies (RR 0.34 CI [0.12,1.01] I2 = 61%) and a lower but insignificant in-hospital mortality in comparison to one-year mortality in studies with mixed etiologies (RR 0.59 [0.11,3.11] I2= 61%). There was a significant improvement in the NYHA grade of the patients one year following pericardiectomy (RR 8.04, CI [5.20,12.45], I2= 0%). The mean postoperative hospital stay and the postoperative ICU stay were calculated and reported in terms of days. The mean postoperative hospital stays in studies with more than 80% of TB cases is 13.34 (10.21, 16.47) with a mean standard deviation of 4.46 (2.87, 6.05). The mean postoperative ICU stay is 1.93 (1.47, 2.39), with a mean standard deviation of 3.26 (2.51, 4.00), and the mean in-hospital mortality is 0.07 (0.02, 0.12). Similarly, the mean postoperative hospital stay in studies with mixed etiologies is 19.40 (11.93, 26.87) with the mean standard deviation of 8.26 (4.21, 12.52). The mean postoperative ICU stay is 3.52 (1.93, 5.10) with the mean standard deviation of 2.34 (1.36, 3.32). The mean in-hospital mortality is 0.06 (0.04, 0.08).

Our paper has several limitations. First, all the studies are retrospective observational studies. Secondly, the sample size is relatively low as the prevalence is mostly in the developing world and the number of studies is limited. Thirdly, there is moderate to high heterogeneity in the studies and therefore caution should be taken while interpreting and generalizing the study. Lastly, there was no sensitivity analysis done. There is significant heterogeneity along with a number of methodological concerns, and therefore generalization of the data should be done with caution and a randomized controlled trial in the future may be beneficial.

## Conclusions

Pericardiectomy is the standard treatment option in patients with symptomatic constrictive tuberculous pericarditis refractory to medical therapy, frequently reported to be performed in middle-aged men. Total pericardiectomy was reported to be the preferable type of pericardiectomy. Our analysis showed decreased mortality in patients with tuberculosis as compared to other etiologies. A significant improvement in NYHA grade was seen postoperatively in patients with constrictive tuberculous pericarditis. A lower pooled mean postoperative hospital stay and postoperative ICU stay but slightly higher in-patient mortality was seen in studies with more than 80% of tuberculosis cases as compared to studies with mixed etiologies. There was a lower but insignificant in-hospital mortality as compared to one-year mortality in patients undergoing pericardiectomy for constrictive pericarditis with mixed etiologies. However, due to significant limitations, the generalization of the study should be done with caution.
